# *Drosophila* Dullard functions as a Mad phosphatase to terminate BMP signaling

**DOI:** 10.1038/srep32269

**Published:** 2016-08-31

**Authors:** Hugo Urrutia, Abigail Aleman, Edward Eivers

**Affiliations:** 1 Department of Biological Sciences California State University Los Angeles, 5151 State University Dr. Los Angeles, CA 90032 USA

## Abstract

Bone morphogenetic proteins (BMPs) are growth factors that provide essential signals for normal embryonic development and adult tissue homeostasis. A key step in initiating BMP signaling is ligand induced phosphorylation of receptor Smads (R-Smads) by type I receptor kinases, while linker phosphorylation of R-Smads has been shown to cause BMP signal termination. Here we present data demonstrating that the phosphatase Dullard is involved in dephosphorylating the *Drosophila* R-Smad, Mad, and is integral in controlling BMP signal duration. We show that a hypomorphic Dullard allele or Dullard knockdown leads to increased Mad phosphorylation levels, while Dullard overexpression resulted in reduced Mad phosphorylations. Co-immunoprecipitation binding assays demonstrate phosphorylated Mad and Dullard physically interact, while mutation of Dullard’s phosphatase domain still allowed Mad-Dullard interactions but abolished its ability to regulate Mad phosphorylations. Finally, we demonstrate that linker and C-terminally phosphorylated Mad can be regulated by one of two terminating mechanisms, degradation by proteasomes or dephosphorylation by the phosphatase Dullard.

Signaling by the transforming growth factor-β (TGF-β) superfamily of ligands is essential for normal embryonic development and tissue homeostasis in the adult. This group of ligands can be sub-divided into two broad families, the TGF-β/Activin and the BMP signaling pathways^1^,^2^. These signal transduction pathways have been shown to be highly conserved in animal species and a group of transcription factors known as R-Smads transduce the intracellular signal of each pathway^1^,^2^. In *Drosophila*, the R-Smad transcription factor Mad (the *Drosophila* homolog of vertebrate R-Smad1/5) transduces BMP signals^3^. To activate this pathway, a BMP dimer must first bind to both its type I (Thickveins, Tkv and Saxophone, Sax) and type II (Punt, Pnt) serine threonine transmembrane receptors. Receptor activation then results in the phosphorylation of Mad in its MH2 domain at two terminal serine residues (serine-valine-serine, SVS, pMad^Cter^)^4^,^5^. Activated Mad then forms a complex with its Co-Smad, Medea (the *Drosophila* homolog of vertebrate Smad4) and accumulates in the nucleus to activate or repress BMP pathway target genes^3^,^4^,^5^,^6^.

Termination of BMP-activated R-Smads can be achieved by a number of different mechanisms. The first example is by hyper-phosphorylating the central linker domain of R-Smad proteins, which initiates their polyubquitinylation and degradation by the proteasome. The kinases which phosphorylate this linker domain and initiate the cellular process of R-Smad degradation are mitogen activated protein kinases (MAPKs), cyclin dependent kinase 8/9 (Cdk8/9) and glycogen synthase kinase 3 (GSK3)^7^,^8^,^9^,^10^,^11^,^12^. A second mechanism to terminate BMP-induced Smad signaling is by dephosphorylating phospho-Smad proteins by a number of different phosphatases. Examples of described phosphatases are protein phosphatase magnesium-dependent 1A (PPM1A), small C-terminal domain phosphatases 1–3 (SCP1-3), protein phosphatase 2A (PP2A) and Myotubularin-related Protein 4 (MTMR4), which terminates BMP signaling by dephosphorylating Smad1/5^13^,^14^,^15^,^16^,^17^,^18^,^19^. *Drosophila* Pyruvate dehydrogenase phosphatase (PDP) and MTMR4/CG3632 are the only Mad phosphatases described to date and both have been shown to specifically dephosphorylate the C-terminal domain of Mad^19^,^20^.

Here we report that the phosphatase Dullard, which functions by cleaving phosphates from phosphorylated serines and threonines, negatively regulates Mad linker and C-terminal phosphorylations. Dullard, a member of the DXDX(T/V) phosphatase family has been previously shown to inhibit BMP signaling in *Xenopus* embryos by causing the dephosphorylation of type I BMP receptors and the ubiquitin-dependent proteasomal degradation of the BMP type II receptors^21^. Two more recent reports in mice and *Drosophila* mutants demonstrate Dullard negatively regulates BMP signaling, however the focus of these studies were to describe genetic interactions between BMP and Dullard, and therefore, insights into the mechanistic interaction of Dullard with BMP remain to be described^22^,^23^. Our findings add new insights into the role Dullard plays during BMP signaling during *Drosophila* development. We demonstrate that Dullard, a perinuclear associated phosphatase operates at the level of the BMP transcription factor dephosphorylation in *Drosophila* cells and tissues. We find that overexpression of Dullard promotes the dephosphorylation of both the linker and C-terminal domains of Mad, while a Dullard hypomorphic allele or Dullard knockdown resulted in the opposite, with elevated Mad phosphorylation levels. We show Dullard physically interacts with Mad and this interaction is dependent on Mad being phosphorylated. We find that mutation of the phosphatase motif in Dullard still allows for Dullard-Mad binding but abolishes its ability to dephosphorylate Mad proteins. In conclusion, we propose that the phosphatase Dullard is a Mad phosphatase and a negative regulator of BMP signaling.

## Results

We began this investigation by carrying out a double stranded RNA interference (dsRNA) screen against an array of *Drosophila* phosphatases to identify proteins which could regulate Mad linker phosphorylations. From this screen we identified Dullard, a member of the DXDX(T/V) serine/threonine phosphatase family as a potential candidate. In support of further pursuing this lead, Liu and colleagues (2011) had reported a genetic interaction between Dullard and BMP signaling in wing imaginal discs and testes^23^, thus we set out to build on their informative study by investigating the mechanistic basis for Dullards involvement in BMP signaling. We utilized a combination of biochemical assays and *in vivo* expression analyses to uncover the role this protein plays in BMP signaling.

**Figure 1:**
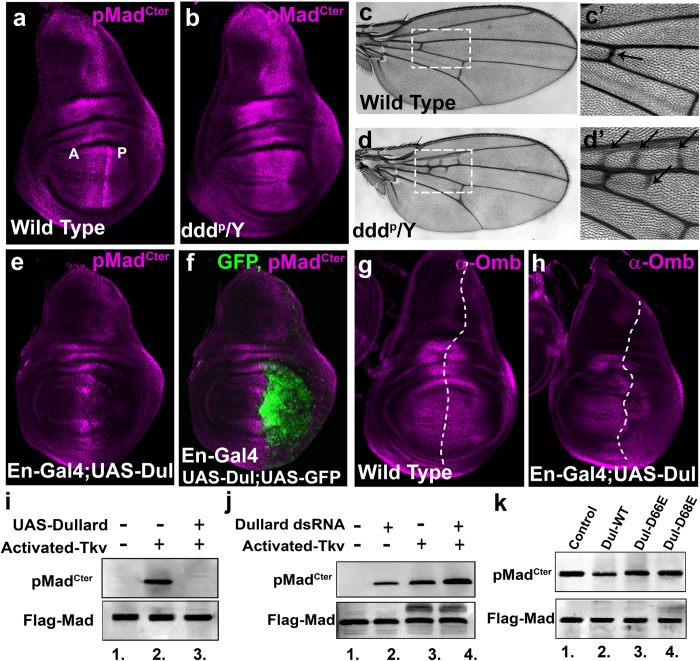
Dullard negatively regulates BMP-activated Mad. (**a**) pMad^Cter^ in wild type wing imaginal disc, anterior compartment (A) to the left, posterior compartment (P) to the right in all wing discs shown (males, n = 15). (**b**) Increased pMad^Cter^ levels in Dullard hypomorph (ddd^P^, a P-element is inserted in the 5′UTR of Dullard) wing imaginal disc (males, n = 31). (**c–c’**) Wild type male adult wing showing normal wing venation (n = 100). Boxed area enlarged in panel c’ showing normal wild type venation surrounding the anterior cross vein (arrow). (**d–d’**) Increased wing venation in hypomorphic Dullard male wings (n = 34/94). Boxed area enlarged in panel d’ shows three ectopic cross veins and one vein spur (arrows) in Dullard hypomorphic wings. (**e,f**) UAS-Dullard driven in the posterior wing compartment using Engrailed-gal4, UAS-GFP results in loss of pMad^Cter^, GFP marker used to highlight cells expressing UAS-Dullard shown in panel f (n = 25). (**g**) Omb protein expression in wild type wing imaginal disc (n = 18). (**h**) UAS-Dullard driven by Engrailed-Gal4 (right of the dashed line) results in a significant decrease in Omb protein expression and reduction in the size of the posterior wing compartment (n = 17) compared to the wild type disc in panel g. (**i**) Dullard overexpression decreases pMad^Cter^ levels S2R+ cells were co-transfected with Flag-Mad, +/− Dullard and +/− Activated-Tkv (to activate BMP signaling). Lysed cells were subjected to western blotting and probed with pMad^Cter^ and Flag (loading control) antibodies (statistical significance when comparing lanes 2 and 3: P < 0.001 using the t-test, n = 3 independent blots measured). (**j**) *Drosophila* S2R+ cells were transfected as in panel i, but cells were treated with +/− Dullard dsRNA. Dullard knockdown results in increased pMad^Cter^ levels (statistical significance when comparing lanes 1 and 2, P = 0.0067; comparing lanes 3 and 4, P = 0.0067, using the t-test, n = 3 independent blots measured). (**k**) Phosphatase inactive Dullard mutants (Dul-D66E and Dul-D68E) failed to dephosphorylate the Mad C-terminal domain compared to wild type Dullard. Mad C-terminal phosphorylation was induced by co-transfecting S2R+ cells with an activated-Tkv receptor in all cases.

Analysis of hypomorphic Dullard mutants (ddd^P^) showed a broad increase in phosphorylated Mad (pMad^Cter^) in wing imaginal discs from male third instar larvae when compared to male wild type wing discs (Fig. 1a,b). This semi lethal fly line was previously described^23^ and has a P-element inserted into the 5′UTR of the Dullard gene. Allowing these hypomorphic male larvae to mature into adults, revealed adult wings with multiple ectopic crossveins (arrows) and spurs associated with longitudinal veins 2 and 3 when compared to wild type wings (Fig. 1c,d’ and Supplementary Table 1), ectopic cross vein tissue can be a sign of increased BMP signaling. In contrast, overexpression of Dullard driven in the posterior wing compartment using Engrailed-Gal4 resulted in loss of pMad^Cter^ (Gal4 expression domain highlighted by green fluorescent protein) and the downstream BMP target gene Optomotor blind (Omb, right of the dashed line) (Fig. 1e–h). These findings in developing larval tissues were supported by western blot analysis of pMad^Cter^ levels in Dullard overexpressing and Dullard knocked down in cultured *Drosophila* S2R+ cells. In these experiments robust phosphorylation of Mad in its C-terminal domain was induced by a constitutively activated-Thickveins (activated-Tkv) receptor as Mad C-terminal phosphorylation was undetectable in the absence of exogenous activation (Fig. 1i, compare lanes 1 and 2). Mad C-terminal phosphorylation levels were found to decrease significantly when Dullard was co-expressed alongside an activated-Tkv receptor (Fig. 1i, compare lanes 2 and 3), while pMad^Cter^ levels were found to increase moderately in cells subjected to Dullard RNAi (Fig. 1j, compare lane 1 to 2 and lane 3 to 4). Interestingly, there was a detectable pMad^Cter^ signal in Dullard dsRNA treated cells which lacked an activated-Tkv receptor, revealing a low level of basal BMP activity does exist in *Drosophila* S2R+ cells (Fig. 1j, compare lanes 1 and 2).

To ensure our dsRNA was specific we used a human Dullard polyclonal antibody to measure total Dullard levels in S2R+ cells. This antibody recognized overexpressed *Drosophila* Dullard protein but failed to recognize endogenous Dullard proteins (Supplementary Fig. S1a, compare lanes 1 and 2). Total Dullard protein levels were found to be reduced in S2R+ cells incubated with our Dullard dsRNA when compared to non dsRNA treated cells, confirming our Dullard dsRNA was indeed specific (Supplementary Fig. S1a, compare lanes 2 and 3). Next we set out to identify the localization of Dullard protein in *Drosophila* S2R+ cells. We found overexpressed Dullard partially co-localized with the nuclear envelope marker, lamin, in addition to localizing to the perinuclear domain of the cell (Supplemental Fig. S1b–e). Finally, in order to test if Dullard’s phosphatase domain, a DXDX(T/V) motif, played a role in affecting Mad C-terminal phosphorylation we mutated either the first essential aspartate (D) residue D66 or second D68 within the catalytic motif into a glutamate residue (D66E and D68E), these mutations have previously been shown to generate a phosphatase-inactive mutant^21^. We found that Dullard phosphatase-inactive mutants failed to affect Mad C-terminal phosphorylations induced by an activated-Tkv receptor (Fig. 1k). From these findings we conclude that perinuclear Dullard is involved in negatively regulating BMP activated Mad as demonstrated by our findings in *Drosophila* larval tissues and western blot analysis.

**Figure 2:**
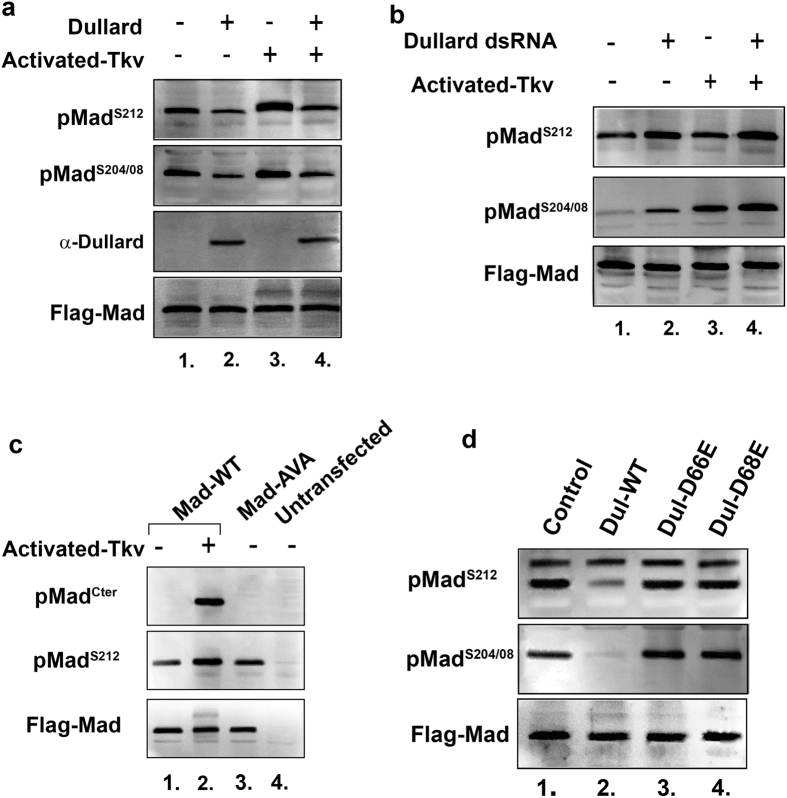
Dullard decreases BMP-dependent and independent Mad linker phosphorylations. In all western blots shown *Drosophila* S2R+ cells were co-transfected with Flag-Mad, +/− Dullard, or +/− Dullard dsRNA and +/− activated-Tkv. (**a**) Dullard overexpression resulted in a decrease in Mad linker phosphorylation levels at serines 212, 208 and 204 (statistical significance when comparing lanes 1 and 2, pMad^S212^ P < 0.001 and pMad^S204/08^ P = 0.004; comparing lanes 3 and 4, pMad^S212^ P < 0.001 and pMad^S204/08^ P < 0.001, using the t-test, n = 3 independent blots measured for each). (**b**) Dullard knockdown resulted in increased levels of Mad linker phosphorylations (statistical significance when comparing lanes 1 and 2, pMad^S212^ P = 0.014 and pMad^S204/08^ P < 0.001; comparing lanes 3 and 4, pMad^S212^ P = 0.014 and pMad^S204/08^ P < 0.001; using the t-test, n = 3 independent blots measured). (**c**) C-terminally mutated Mad proteins (Mad-AVA) are linker phosphorylated. These blots demonstrated that Mad-AVA can be linker phosphorylated in the absence of C-terminal activation by the BMP pathway. (**d**) Phosphatase inactive Dullard mutants (Dul-D66E and Dul-D68E) failed to dephosphorylate the Mad linker domain compared to wild type Dullard.

We next investigated if Dullard manipulation in cultured cells could affect Mad linker phosphorylations at serines 212, 208 and 204. These sites had been previously shown to be sequentially phosphorylated by Cdk8 (phosphorylates serine 212) and Shaggy (phosphorylates serines 204 and 208) in response to Mad C-terminal phosphorylation^12^. Mad linker phosphorylations were detectable by western blotting even in the absence of an exogenous BMP pathway activator and overexpression of Dullard significantly reduced these linker phosphorylations (Fig. 2a, compare lanes 1 and 2). Next to more robustly activate the BMP pathway we used a constitutively activated-Tkv receptor, this resulted in significantly increased Mad linker phosphorylations over basal levels at serines 212, 208 and 204 (Fig. 2a, compare lanes 1 and 3). When Dullard was co-expressed with an activated-Tkv receptor, Mad linker phosphorylation levels were once again found to decrease (Fig. 2a, compare lanes 3 and 4). In contrast Dullard knockdown in cells lacking or expressing an activated-Tkv receptor resulted in increased Mad linker phosphorylation levels when compared to non-dsRNA situations (Fig. 2b). As mentioned above, increased Mad linker phosphorylation levels occurred in response to Mad C-terminal phosphorylation, however, it has previously been shown that Mad can also be phosphorylated in its linker domain in the absence of C-terminal phosphorylation^24^,^25^,^26^. To determine whether Dullard could regulate this population of linker phosphorylated Mad we used a Mad-AVA mutant in which its two terminal serines were mutated into alanines (A), thus completely bypassing BMP receptor input. In the absence of an activated-Tkv receptor we found that Mad-AVA is phosphorylated at all three linker serines (Fig. 2c, lane 3 and data not shown) and these phospho-forms of Mad co-localized to both the cytoplasmic and nuclear compartments of the cell (Supplemental Fig. S2a,b). In Dullard overexpression experiments we find Dullard can effectively reduce Mad-AVA linker phosphorylation (Supplementary Fig. S2c) while Dullard knockdown caused an increase in Mad-AVA linker phosphorylation levels (Supplementary Fig. S2d). Finally, we investigated the effect Dullard catalytic domain mutants (D66E and D68E) had on Mad linker phosphorylation levels. We found that mutation of these sites inhibited Dullard’s ability to affect basal phosphorylation of the Mad linker domain when compared to cell extracts expressing wild type Dullard (Fig. 2d). From these data we demonstrate that Dullard plays a key role in negatively regulating Mad linker phosphorylations induced by the BMP pathway or independent of BMP signaling input.

**Figure 3:**
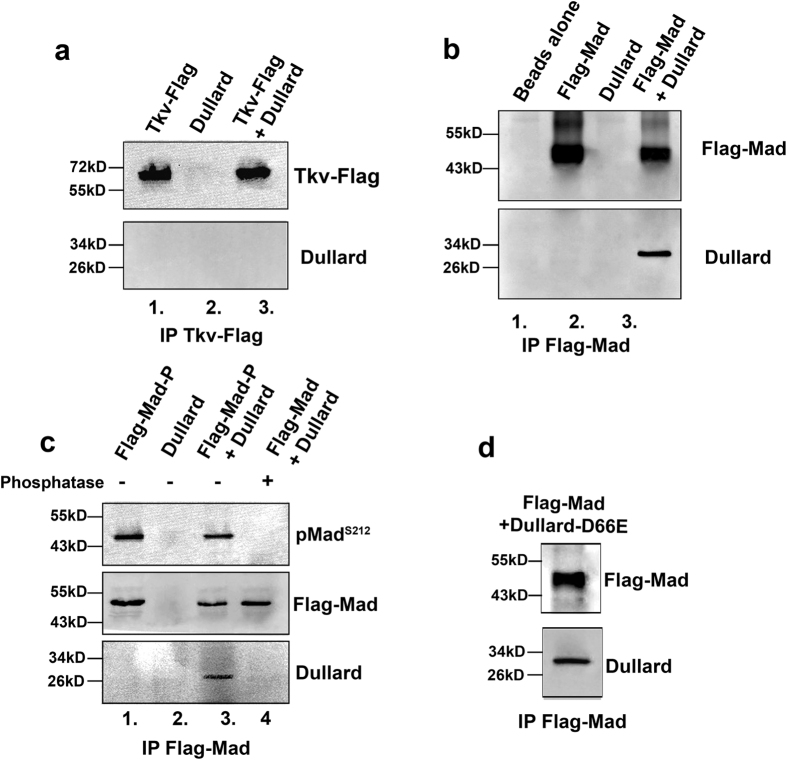
Dullard physically interacts with Mad. (**a**) S2R+ cells were co-transfected with Tkv-Flag and Dullard. Tkv-Flag was immunoprecipitated (IP) with anti-flag beads and then subjected to western blotting to evaluate if Tkv and Dullard interact. Results demonstrate Tkv and Dullard do not physically interact. (**b**) S2R+ cells were co-transfected with Flag-Mad and Dullard. Flag-Mad was immunoprecipitated (IP) with anti-flag beads and then subjected to western blotting to evaluate if Mad and Dullard interact. Results demonstrate Dullard and Mad proteins do physically interact. (**c**) Treatment of Flag-Mad lysates with calf intestinal phosphatase dephosphorylated Mad (shown by using pMad^S212^ antibody) compared to untreated lysates, compare lanes 3 and 4. Dephosphorylated Mad failed to bind Dullard compared to phosphorylated Mad, compare lanes 3 and 4. (**d**) S2R+ cells were co-transfected with Flag-Mad and Dullard-D66E. Flag-Mad was immunoprecipitated (IP) with anti-flag beads and then subjected to western blotting to investigate if both proteins interact. Results demonstrate Dullard-D66E and Flag-Mad proteins do physically interact. All western blots presented were repeated at least 2 times.

We next investigated if there is a physical interaction between Dullard and either the BMP type I receptor Tkv or the transcription factor Mad by co-immunoprecipitation (Co-IP) assay. Tkv-Flag and Dullard or Flag-Mad and Dullard were co-transfected into *Drosophila* S2R+ cells and lysed 48 hours later. We found that Dullard failed to co-immunoprecipitate with Tkv-Flag but interacted robustly with Flag-Mad, thus demonstrating an interaction between Mad and Dullard proteins (Fig. 3a,b, inputs are shown in Supplementary Fig. S3a,b). In addition, we carried out a reverse Co-IP experiment and demonstrated that Flag-Dullard could pulldown total Mad (Supplementary Fig. 3c,d). Next we investigated if Mad phosphorylation was a requirement for Dullard-Mad interactions. In this assay Flag-Mad and Dullard were transfected into separate culture wells, cells were lysed 48 hours later and Flag-Mad lysate samples were treated with calf intestinal alkaline phosphatase for 1 hour at 37 °C to dephosphorylate Mad. Dullard and Flag-Mad lysates were subsequently mixed and subjected to Co-IP. This assay revealed no binding between dephosphorylated Flag-Mad and Dullard when compared to binding between phosphorylated Flag-Mad and Dullard (Fig. 3c, compare lanes 3 and 4, inputs are shown in Supplemental information Fig. S3e). To test if the Dullard catalytic domain was essential for Mad-Dullard interactions we repeated the Co-IP assay using Flag-Mad but now used the phosphatase inactive Dullard mutant (D66E). From this assay we found that the interaction between Dullard and Flag-Mad was not dependent on a functional phosphatase domain as the catalytic inactive mutants still formed a complex with Flag-Mad (Fig. 3d, inputs are shown in Supplemental information Fig. S3f). Next we tested if Medea, a co-Smad (the homolog of vertebrate Smad4), was required for Dullards role in regulating Mad phosphorylations. In this experiment we overexpressed Dullard in the presence or absence of Medea dsRNA^25^. We found that when Medea was depleted from S2R+ cells it had no effect on Dullards ability to decrease Mad C-terminal or linker phosphorylations (Supplemental Fig. 4). From these data we conclude that phosphorylated Mad and Dullard interact and that Medea is not required for Dullards role in regulating Mad phosphorylations.

**Figure 4:**
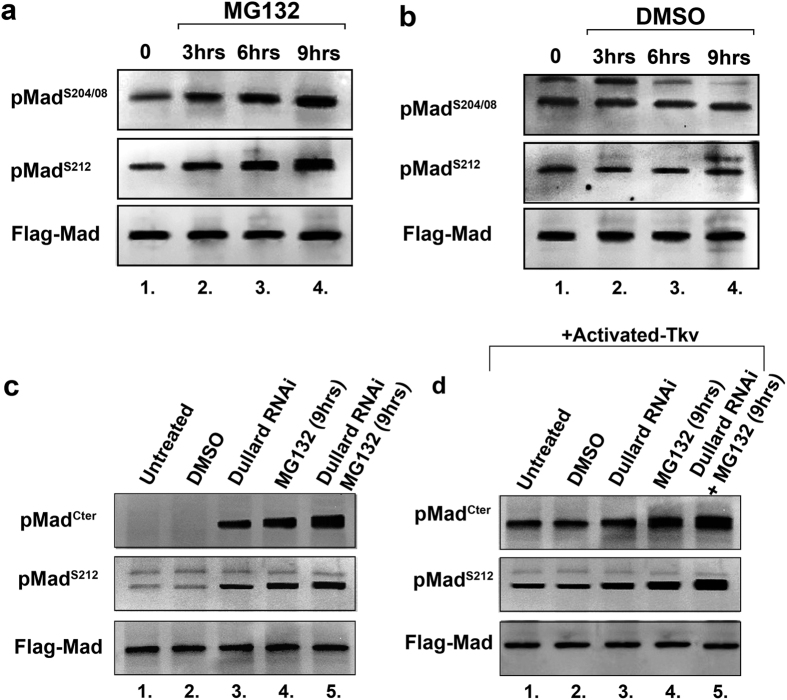
Termination of BMP signaling by dephosphorylation and/or degradation of phospho-Mad. (**a**) 9 hour time course assay showing stabilization of linker phosphorylated Mad using the proteasomal inhibitor MG2132 (MG132 was resuspended in DMSO). (**b**) Treatment of S2R+ cells with DMSO alone over 9 hours shows it has no effect on Mad linker phosphorylation levels. (**c**) Mad phosphorylations were stabilized by Dullard knockdown or proteasomal inhibition (MG132). Strongest stabilization is found when both Dullard and the proteasome were inhibited. Note Mad C-terminal phosphorylation is not detectable in the absence of an activated-Tkv receptor, however, C-terminal phosphorylation becomes evident either by knocking down the phosphatase Dullard or inhibiting the proteasome by MG132. (**d**) Activated-Tkv induced C-terminal and linker phosphorylated Mad was strongly stabilized by Dullard knockdown and/or proteasomal inhibition compared to control untreated or DMSO treated cells.

Vertebrate Smad1/5 linker phosphorylations have previously been shown to cause BMP signal termination by causing Smad to be degraded by the proteasome^9^,^10^,^11^. To demonstrate the role the proteasome may play in controlling phospho-Mad levels in *Drosophila* cells we ran a time course assay using the proteasomal inhibitor MG132. S2R+ cells transfected with Flag-Mad were treated with the MG132 inhibitor for 0, 3, 6, and 9 hours. Western blot analysis of these cell extracts revealed increasing levels of Mad linker phosphorylation over time in the presence of the proteasomal inhibitor and absence of an activated-Tkv receptor (Fig. 4a). Bulk Flag-Mad or total Mad levels were found not change in the presence of the proteasomal inhibitor, this is in agreement with other Smad1 studies which have reported only a tiny fraction of total Smad1 is phosphorylated in cells, with the majority remaining as a large reservoir of inactive Smad1^9^,^27^. In control DMSO treated cells, a flat steady state of basal Mad linker phosphorylation levels was found over the 9 hour period (Fig. 4b). Next we treated S2R+ cells with either Dullard RNAi, the MG132 inhibitor or both together and analyzed Mad linker and C-terminal phosphorylations in the absence of an activated-Tkv receptor. Treatment of S2R+ cells with either Dullard dsRNA or MG132 inhibitor increased linker and C-terminal phosphorylations over control situations, while treatment of cells with both Dullard dsRNAi and MG132 together strongly stabilized linker and C-terminal phosphorylations over either treatment situation alone (Fig. 4c). As was seen in Fig. 1j, in the absence of exogenous BMP pathway activation (such as activated-Tkv) no C-terminal phosphorylation was detected by western blot in S2R+ cells, however, treatment of these cells with Dullard dsRNA or MG132 allows Mad C-terminal levels to be detected (Fig. 4c, lanes 3–5). Similar phospho-Mad stabilization was detected when Flag-Mad cells were co-transfected with an activated Tkv receptor and treated with Dullard dsRNA and MG132 or both together (Fig. 4d). Finally, we repeated the above MG132 time course experiment using Flag-Mad-AVA, which is linker phosphorylated in the absence of BMP pathway stimulation and found linker phosphorylated Mad-AVA to be strongly stabilized over a 9 hour treatment period (Supplemental Fig. S5a,b). Choosing the 9 hour MG132 treatment time point we found Mad-AVA linker phosphorylations were strongly stabilized by MG132 and/or Dullard dsRNA treatment and no effect on Mad-AVA linker phosphorylation was noted when S2R+ cells were treated with Tkv dsRNA (Supplemental Fig. S5c). These data demonstrate that this BMP-independent population of linker phosphorylated Mad can also be regulated by proteasomal degradation and dephosphorylation by the phosphatase Dullard.

In conclusion, our findings have identified a new regulator of BMP signaling pathway in *Drosophila*. We demonstrated that the phosphatase Dullard is capable of negatively regulating Mad C-terminal and linker phosphorylations, by directly binding to phospho-Mad and that proteasomal degradation and dephosphorylation are two possible mechanisms of terminating BMP signaling.

## Discussion

Rigorous control of the BMP signaling pathway is essential for normal growth and development. Smad/Mad linker phosphorylations which result in protein degradation have previously been shown to be an important process in controlling the duration of BMP signals^9^,^10^,^11^,^12^. Here we propose that Mad linker and C-terminal dephosphorylation is an alternative mechanism of regulating BMP signal duration in *Drosophila*. Our findings adds a new phosphatase to the growing number which have already been shown to dephosphorylate vertebrate Smad proteins. Several Smad1/5 phosphatases have been described, these include SCP1-3, PPM1A, MTMR4, PP2A, and the *Drosophila* Mad phosphatase, PDP^13^,^14^,^15^,^16^,^17^,^18^,^19^,^20^. These phosphatases have been shown to terminate BMP signals by dephosphorylating the BMP-activated C-terminal domain of Mad/Smad1/5 or by dephosphorylating both the C-terminal and linker domains of vertebrate Smad1/5. In this study we set out with the goal of identifying novel modulators of linker phosphorylated Mad and this lead us to discover Dullard as a negative regulator of Mad phosphorylations in *Drosophila*. Here we report that the actions of Dullard’s catalytic phosphatase domain can negatively regulate BMP signaling by dephosphorylating Mad’s C-terminal and linker domains, and mutation of Dullard’s catalytic domain inhibited its ability to regulate these phosphorylations. In addition, we demonstrated that Dullard can also regulate Mad linker phosphorylations independent of BMP signaling input (Mad C-terminal phosphorylation). We then provide evidence that Dullard physically interacts with Mad and that phosphorylation of Mad is required for Dullard-Mad interactions. Finally, we demonstrated that phosphorylated Mad proteins (C-terminal and linker) can undergo one of two signal terminating events, degradation by proteasomes initiated by linker phosphorylation or Mad protein recycling by the dephosphorylating activity of Dullard. The finding that Dullard is capable of dephosphorylating both the linker and C-terminal domains of Mad demonstrates that Dullard is not selective in what protein domains it dephosphorylates and that it may also dephosphorylate other known regulatory phosphorylations in *Drosophila* Mad such as phosphorylated serine 25 and threonine 312^28^,^29^.

The phosphatase Dullard was first described as a novel gene required for *Xenopus* neural development and subsequently shown to be negative regulator of BMP signaling by causing the dephosphorylation of type I BMP receptors and the ubiquitin-dependent proteasomal degradation of type II BMP receptors^21^,^30^. More recent studies in postnatal mouse nephron development^22^ and wing vein formation in *Drosophila*^23^ also supported the finding that Dullard was a negative regulator of the BMP pathway. However, in these two latter studies a mechanistic link to the BMP pathway was not demonstrated as they focused on a thorough analysis of genetic interactions between Dullard and the BMP pathway. Our biochemical data presented here also support the premise that Dullard is a negative regulator of the BMP signaling pathway acting at the level of the BMP transcription factor Mad in the perinuclear and nuclear envelope regions of the cell. Our study presented here focuses primarily on Mad C-terminal and linker dephosphorylation and further biochemical and *in vivo* studies would need to be carried out to investigate if *Drosophila* Dullard, like that found for *Xenopus* Dullard^21^, has additional regulatory interactions with the BMP pathway, such as promoting the proteasomal degradation of the BMP type II receptor Pnt and the dephosphorylation of the BMP type I receptors Sax and Tkv.

Dullard’s task in regulating Mad phosphorylations may extend beyond its role in negatively regulating the BMP signaling pathway, recently *Drosophila* Mad has been shown to have multiple BMP-independent functions. For example it has been shown that unphosphorylated Mad is part of the Wingless transcriptional complex of Pangolin and Armadillo and that linker or C-terminal phosphorylation of Mad could inhibit Wingless signaling^25^. As Dullard can dephosphorylate both domains of Mad independent it could potentially enhance Wingless signaling by increasing unphosphorylated Mad available to interact with the transcriptional complex of Pangolin and Armadillo. Another recently reported BMP-independent role for Mad describes how Mad linker phosphorylation by Shaggy/Zw3 functions to prevent self-renewal of sensory organ precursors expressing Senseless^26^. In this case Dullards dephosphorylating activity may act to promote self-renewal of this cell type during early development.

Our findings presented here describe an additional mechanism of terminating BMP signaling in *Drosophila*. Over the last number of years a lot of effort was focused on understanding how linker phosphorylated Mad/Smad1/5 could terminate BMP signaling. The finding that Dullard can dephosphorylate Mad in its linker and C-terminal domains has a number of significant outcomes for BMP signaling in *Drosophila*. Firstly, we propose dephosphorylation of phosphorylated Mad proteins terminates BMP signaling and secondly that dephosphorylated Mad may act as a recycling mechanism where Mad may once again interact with membrane bound BMP receptors for additional rounds of signaling. In conclusion, we propose that the phosphatase Dullard is a Mad phosphatase which controls BMP signal duration.

## Methods

### Drosophila strains

Canton S (Bloomington #1), Engrailed-Gal4, UAS-GFP (Bloomington #25752), Dullard flies: ddd^P^, UAS-Dullard. All *Drosophila* crosses were carried out at room temperature 25 °C.

### Wing disc fixation

Wing imaginal discs were dissected out of third instar larva in Schneider’s media. Discs were fixed in Browers solution for 30 minutes on ice and rinsed using PBS/0.02% Triton X-100.

### Immunostaining and imaging

Wing disc immunostainings were carried out using standard protocols. Wing discs and/or cultured S2R+ cells were immunostained using the following primary antibodies: pMad^Cter^, 1:1000 (E. Laufer); anti-Omb 1:700 (V. sander); anti-Lamin (ADL67, Hybridoma bank), and pMad^S212^, 1:1000 (E. De Robertis). The following secondary antibodies (Jackson Laboratories) were used: anti-mouse Cy3 conjugated antibody 1:1000, anti-mouse 488 conjugated antibody 1:1000, anti-rabbit Cy3 conjugated antibody 1:1000, anti-rabbit 488 conjugated antibody, 1:1000. All tissues were placed in DAPI-containing Vectashield (Vector) and mounted on glass slides. Fluorescent imaging was carried out using a Zeiss Apotome microscope and accompanying Zeiss software (pseudo-coloring). Wing imaginal discs were imaged at 10x magnification and cultured cells imaged at 63x magnification.

### Western blotting

All tissue samples were lysed in RIPA buffer containing phosphatase and protease inhibitors unless otherwise stated. All western blotting was carried out using standard protocols. Primary antibodies were used at the following concentrations, pMad^Cter^ (Cell Signaling), 1:1000; pMad^212^ (E. De Robertis), 1:1000; pMad^204/208^ (E. De Robertis), 1:1000; anti-Flag (Sigma), 1:1000, anti-β-Tubulin (E7-c, Hybridoma bank), 1:1000; anti-Dullard (Sigma), 1:1000; anti-Mad (Newfeld)^31^ 1:1000. Secondary antibodies (Thermo Scientific) anti-mouse, anti-rabbit and anti guinea pig 1:1000.

### Cell Culture, dsRNAi treatment and transfection

*Drosophila* S2R+ cells were cultured in Schneider’s media, containing 10% FBS. Growing and culturing of cells followed *Drosophila* Genomic Resource Center protocols (https://dgrs.cgb.indiana.edu/Protocols?tab=cells). S2R+ cells were transfected with plasmid DNAs 24 hours after dsRNAi treatment following the Qiagen Effectene protocol. DNAs used in this study were UAST-Mad-WT (DeRobertis), UAST-Mad-AVA (DeRobertis) and pAWF-Tkv-Flag (K. Wharton). Dullard was PCR amplified using *Drosophila* cDNA and cloned into the pAc5.1 vector using Kpn1 and Xba1.

### Immunoprecipitation

Cells were transfected with the following plasmids: UAS-Flag-Mad, UAS-Mad-AVA, pAC-Dullard or pAWF-Tkv-Flag. All cell samples were lysed in RIPA buffer containing phosphatase and protease inhibitors. EZview Red ANTI-FLAG M2 Affinity gel beads (Sigma) were used following standard protocols. All subsequent western blotting was carried out using standard protocols. Primary antibodies were used at the following concentrations, anti-Flag (Sigma), 1:1000; anti-Dullard (Sigma), 1:1000. Secondary antibodies (Thermo Scientific) anti-mouse and anti-rabbit, 1:1000.

### Phosphorylation-dephosphorylation assays

*Drosophila* S2R+ cells were transfected with UAS-Flag-Mad and pAC-Dullard plasmids. The cells were harvested 48 hrs later by pipetting and centrifugation in 150 μl of RIPA buffer (25 mM Tris HCl pH 7.6, 150 mM NaCl, 1% NP-40 without SDS) containing protease inhibitors. Lysates were then incubated in the presence or absence of 60 units of calf intestinal alkaline phosphatase (New England Biolabs) for 60 min at 37 °C. All samples were subjected to binding assays and western blotting as described above.

## Additional Information

**How to cite this article**: Urrutia, H. *et al*. *Drosophila* Dullard functions as a Mad phosphatase to terminate BMP signaling. *Sci. Rep.* **6**, 32269; doi: 10.1038/srep32269 (2016).

## Supplementary Material

Supplementary Information
