# Corrigendum: *Drosophila* Dullard functions as a Mad phosphatase to terminate BMP signaling

**DOI:** 10.1038/srep46923

**Published:** 2017-12-22

**Authors:** Hugo Urrutia, Abigail Aleman, Edward Eivers

Scientific Reports 6: Article number: 32269; 10.1038/srep32269 published online: 08 31 2016; updated: 12 22 2017.

This Article contains errors in Figure 4, where the blot for pMad^S212^ in panel 4c was unintentionally swapped with the pMad^Cter^ blot from panel 4d. In addition, Figure S2 in the original version of the Supplementary Information was incorrect, where the blots for Flag-Mad-AVA were unintentionally swapped between panels S2c and S2d.

The correct Figure 4 appears below and the Supplementary Information file has now been replaced. In the revised version, Supplementary Figure 2 has been corrected and the unprocessed images of full-length blots for all blots presented in this Article are also included as Supplementary Figures 6–14.

Additionally, in the Methods section, the following information had been omitted and now is added here for clarity:

“Western blots were imaged using a versa doc imaging system 5000MP (Bio-Rad). In a number of cases the same western blot was stripped and re-probed. The stripped and re-probed blots include;

Figure 1j pMad^Cter^ is a stripped and re-probed blot of figure 2b pMad^S212^.

Figure 2a Dullard is a stripped and re-probed blot of figure 2a Flag-Mad. 

Figure 2b The pMad^S204/08^ is a stripped and re-probed blot of figure 1j, pMad^Cter^.

Figure 4a The pMad^S212^ is a stripped and re-probed blot of figure 4a Flag-Mad. 

Figure 4b The pMad^S212^ blot is a stripped and re-probed blot of panel 4b Flag-Mad.

Figure 4c pMad^S212^blot is a stripped and reprobed blot of 4c flag-Mad.

Figure 4d pMad^Cter^ is a stripped and reprobed blot of 4d pMad^S212^.

Supplemental Fig. 2d pMad^S204/08^ is a stripped and re-probed blot of Supplemental Fig. 2d Flag-Mad AVA

Supplemental Fig. 4 pMad^Cter^ is a stripped and re-probed blot of pMad^S212^”.

The conclusions of the Article remain unchanged. The authors apologise for the errors.

## Figures and Tables

**Figure 1:**
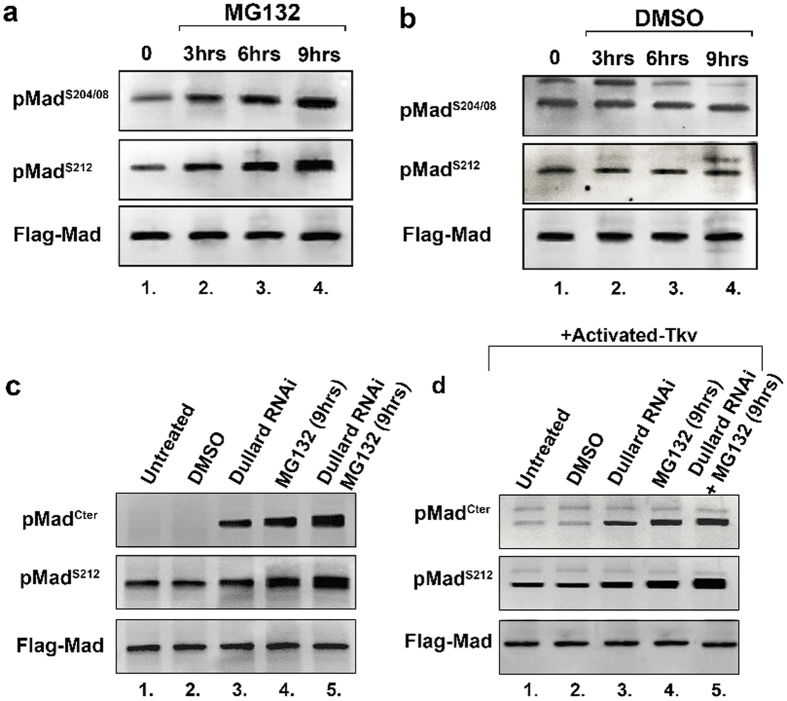
Spicule types and spicule formation in S. ciliatum.

