# Supplementary material for: Drosophila Dullard functions as a Mad phosphatase to terminate BMP signaling
Source: Sci Rep. 2016 Aug 31;6:32269. doi: 10.1038/srep32269 (PMC5006046; doi:10.1038/srep32269)
Supplement: Supplementary Information [file srep32269-s1.pdf]

Supplemental Materials for

Drosophila Dullard functions as a Mad phosphatase to terminate BMP signaling

Hugo Urrutia[a†], Abigail Aleman[a†] and Edward Eivers[a*]

Author Affiliations:

[a] Department of Biological Sciences
California State University Los Angeles
5151 State University Dr.
Los Angeles, CA 90032
USA

[†]Both authors contributed equally to this work

*To whom correspondence should be addressed. E-mail: eeivers@calstatela.edu

Tel: (323) 343 2075; Fax: (323) 343 6451

This PDF file includes:
Supplemental Fig. S1: Dullard localization in S2R+ cells
Supplemental Fig. S2. Dullard regulates linker phosphorylated Mad not induced by the BMP pathway
Supplemental Fig. S3. Whole lysate inputs and Flag-Dullard and Mad Co-IP
Supplemental Fig. S4. Medea knockdown does not affect Dullards regulation of Mad phosphorylations
Supplemental Fig. S5. Dullard regulation of Mad-AVA linker phosphorylations
Supplemental Table S1

SUPPLEMENTAL FIGURE LEGENDS

Supplemental Fig. S1. Dullard localization in S2R+ cells. (a) Western blot demonstrating the specificity of our Dullard dsRNA. Dullard dsRNA treatment of S2R+ cells reduces total amounts of overexpressed Dullard protein (compare lanes 2 and 3). (b-d) Dullard localizes to the nuclear envelope (marked by Lamin, panel e) and perinuclear region (cytoplasmic region surrounding the nucleus) of the cell.

Supplemental Fig. S2. Dullard regulates linker phosphorylated Mad not induced by the BMP pathway (a-b) Linker phosphorylated Mad-AVA protein co-localizes to the cytoplasmic and nuclear regions of the cell. Cells were stained with pMad^{S212} (red) and DAPI (blue, nucleus). Untransfected cells display little or no pMad^{S212} staining (arrows). (c) Dullard overexpression decreased Mad-AVA linker phosphorylations. (d) Dullard knockdown resulted in an increase in Mad-AVA linker phosphorylation levels compared to non dsRNA treated cells. Flag-Mad was used as a loading control, all western blots were repeated at least 2 times.

Supplemental Fig. S3. Whole lysate inputs and Flag-Dullard and Mad Co-IP. (a-b) Whole lysates (10% inputs) probed with anti-Flag (Tkv or Mad) and anti-Dullard antibodies (c) Whole lysates (10% inputs) probed with Flag (Dullard) and total Mad. (d) S2R+ cells were co-transfected with Flag-Dullard and pAC-Mad. Flag-Dullard was immunoprecipitated (IP) with anti-flag beads and then subjected to western blotting to evaluate if Dullard and total Mad interact. Results demonstrate Dullard and Mad proteins do physically interact. (e) Whole lysates probed with anti-Flag, anti-Dullard and pMad^{S212} before phospho-Mad-Dullard immunoprecipitation assay (10% inputs). (f) Immunoblots probed with anti-Flag and anti-Dullard antibodies before Mad-Dullard D66E immunoprecipitation assay (10% inputs).

Supplemental Fig. S4. Medea knockdown does not affect Dullards regulation of Mad phosphorylations. Overexpression of Dullard decreases Mad phosphorylation levels. Medea knockdown using dsRNA does not inhibit Dullards ability to dephosphorylate Mad proteins.

Supplemental Fig. S5. Dullard regulation of Mad-AVA linker phosphorylations. (a) Time course showing stabilization of linker phosphorylated Flag-Mad-AVA using the proteasomal inhibitor MG2132 (MG132 was resuspended in DMSO). (b) Treatment of S2R+ cells with DMSO over 9 hours shows it has no effect on Flag-Mad-AVA linker phosphorylation levels. (c) Mad-AVA phosphorylations were stabilized by Dullard dsRNA and/or MG132 treatment. Tkv dsRNA had no effect on Mad-AVA linker phosphorylation levels.

Supplemental Table S1: Percentages of Dullard hypomorphic adult male flies having ectopic crossveins compared to wild type adult male flies

Genotype	Normal cross veins (%)	Wings with ectopic Crossveins %	Total number of wing counted
Wild type	100	0	100
ddd^{P}/Y	64	36	94

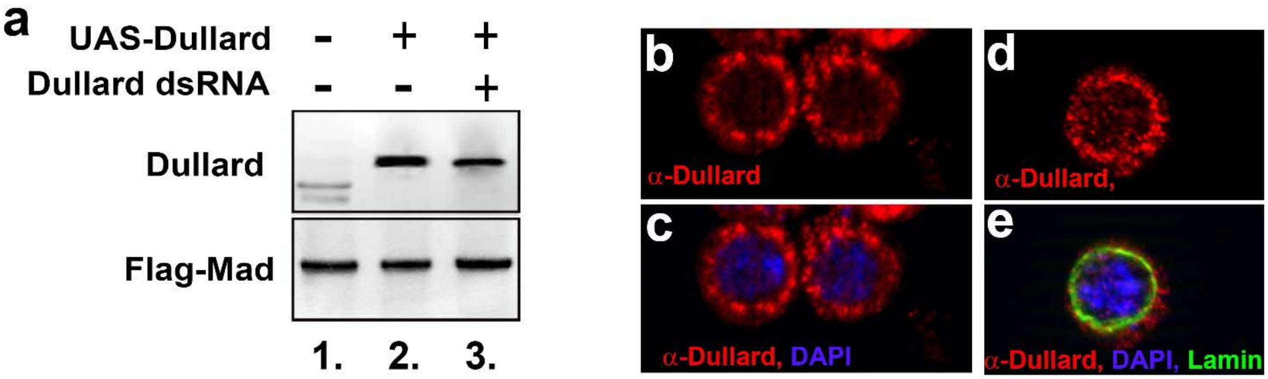

Supplemental Fig. S1

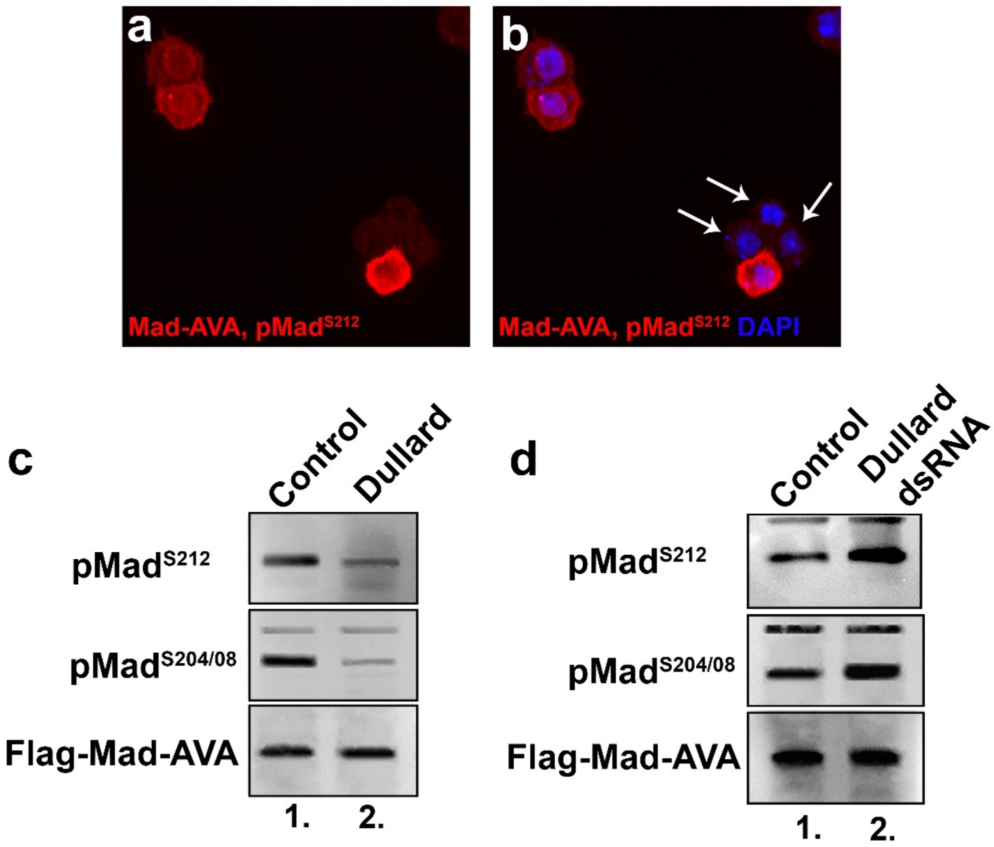

Supplemental Fig. S2

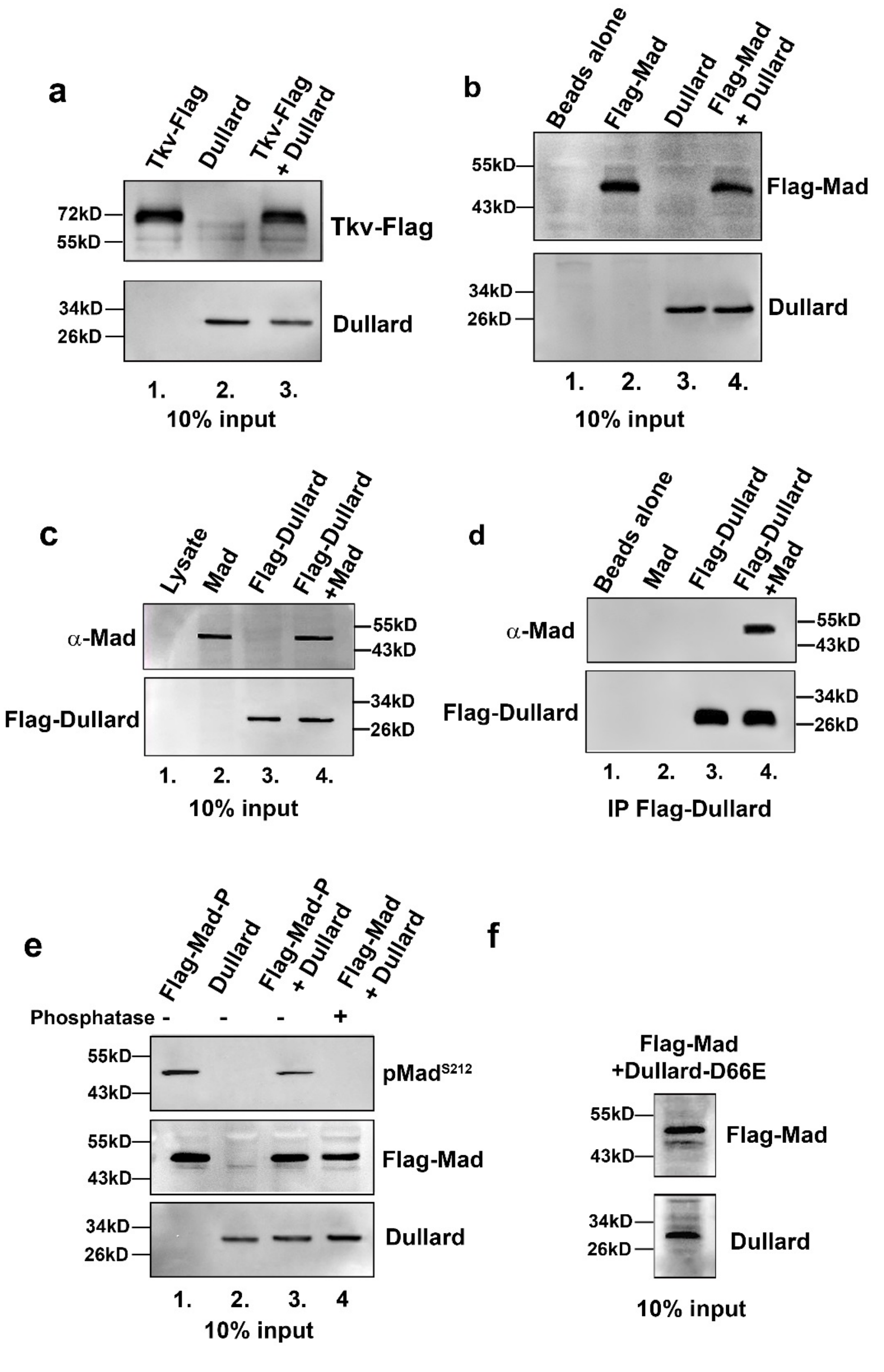

Supplemental Fig. S3

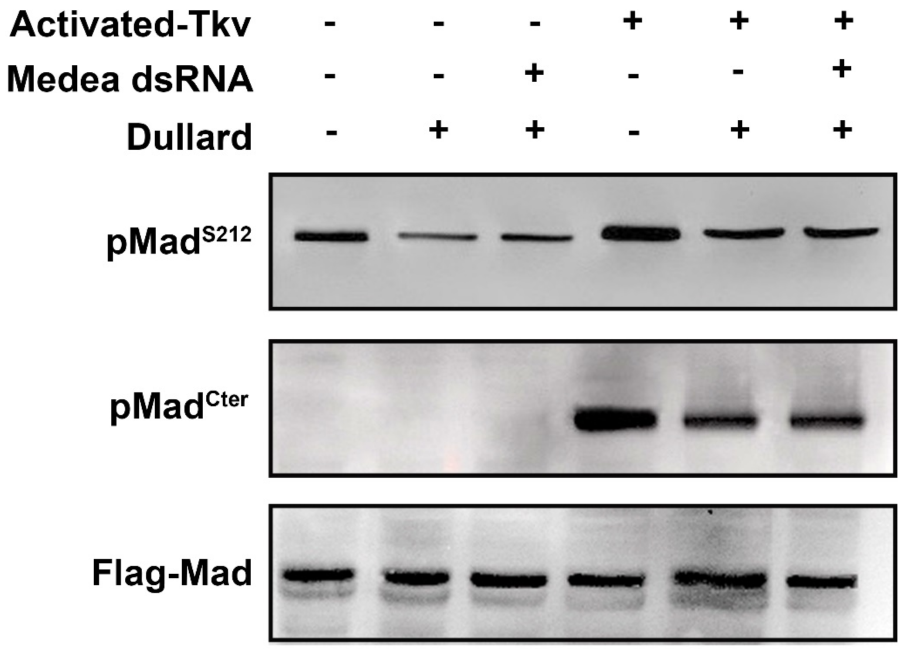

Supplemental Fig. S4

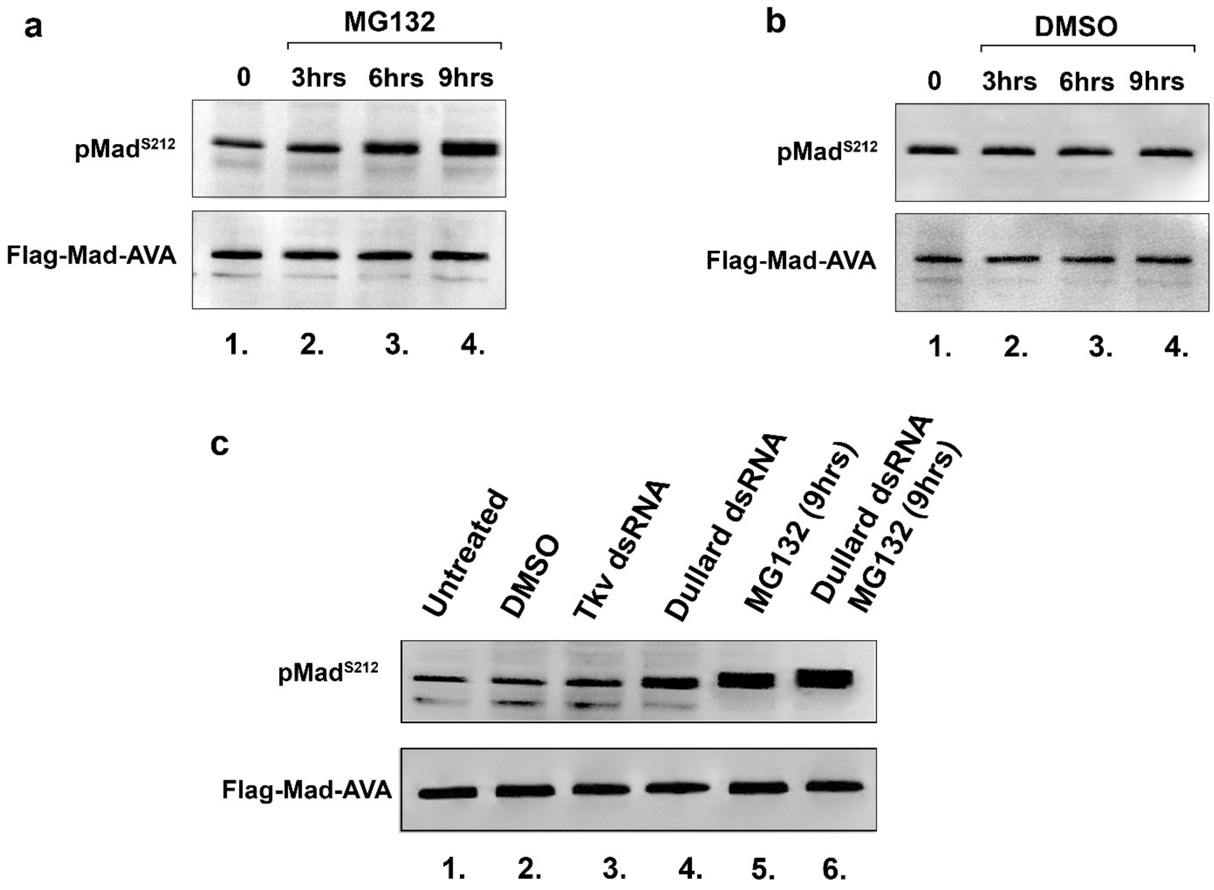

Supplemental Fig. S5
